# Maintenance of neuronal fate and transcriptional identity

**DOI:** 10.1242/bio.059953

**Published:** 2023-06-05

**Authors:** Gabriel N. Aughey

**Affiliations:** Queen Square Institute of Neurology, Department of Clinical and Experimental Epilepsy, University College London, London WC1N 3BG, UK

**Keywords:** Development, Epigenetics, Neurobiology, Neurodegeneration, Transcription

## Abstract

The processes that drive naive multipotent stem cells towards fully differentiated fates are increasingly well understood. However, once differentiated, the mechanisms and molecular factors involved in maintaining differentiated states and associated transcriptomes are less well studied. Neurons are a post-mitotic cell-type with highly specialised functions that largely lack the capacity for renewal. Therefore, neuronal cell identities and the transcriptional states that underpin them are locked into place by active mechanisms that prevent lineage reversion/dedifferentiation and repress cell cycling. Furthermore, individual neurons may be very long-lived, so these mechanisms must be sufficient to ensure the fidelity of neuronal transcriptomes over long time periods. This Review aims to provide an overview of recent progress in understanding how neuronal cell fate and associated gene expression are maintained and the transcriptional regulators that are involved. Maintenance of neuronal fate and subtype specification are discussed, as well as the activating and repressive mechanisms involved. The relevance of these processes to disease states, such as brain cancers and neurodegeneration is outlined. Finally, outstanding questions and hypotheses in this field are proposed.

## Teaser

**Summary:** This Review explores recent progress in understanding the mechanisms that maintain neuronal cell fate and gene expression, discussing the activating and repressive factors involved, relevance to disease, and outstanding questions.

## Introduction

Multicellular organisms are comprised of multiple highly specialised cell-types that confer unique biological functions. These cells are derived via the process of differentiation from multipotent precursors. This is (on the whole) a uni-directional process; differentiated cells once derived, rarely revert to more naive states. Differentiation requires the coordination of transcriptional programmes that promote the expression of genes required for a cell's specific biochemical activities within the context of the functioning organism. These programmes involve the timely activation of required genes, as well as repression of inappropriate gene expression. The processes required to promote distinct cell fates are well-studied. However, once differentiation has been established, there is less appreciation for the subsequent mechanisms that maintain cells in their mature states.

Differentiated neurons are post-mitotic cells; having exited the cell cycle, they lack the ability to further divide. Many organisms’ nervous systems have limited regenerative capacity, i.e. they are unable to produce new neurons in adulthood, meaning that neurons born in their early life-stages persist for the entire lifespan without replacement. This means that the gene expression programmes that are required for coordinating nervous system function must be maintained over years or decades (or potentially even centuries in extreme cases; Nielsen et al., 2016).

This Review aims to provide an overview of our current understanding of the mechanisms that maintain neuronal cell states by preventing lineage reversion or developmental stage inappropriate gene expression as well as ensuring the stable expression of genes that confer neuron-specific functions which together define the identity of fully differentiated, post-mitotic neuronal states. The implications for these processes in diseases such as Alzheimer's disease and cancer are discussed and outstanding questions in the field highlighted.

## Transcription factors responsible for maintaining overall neuronal identity

**Fig. 1.**
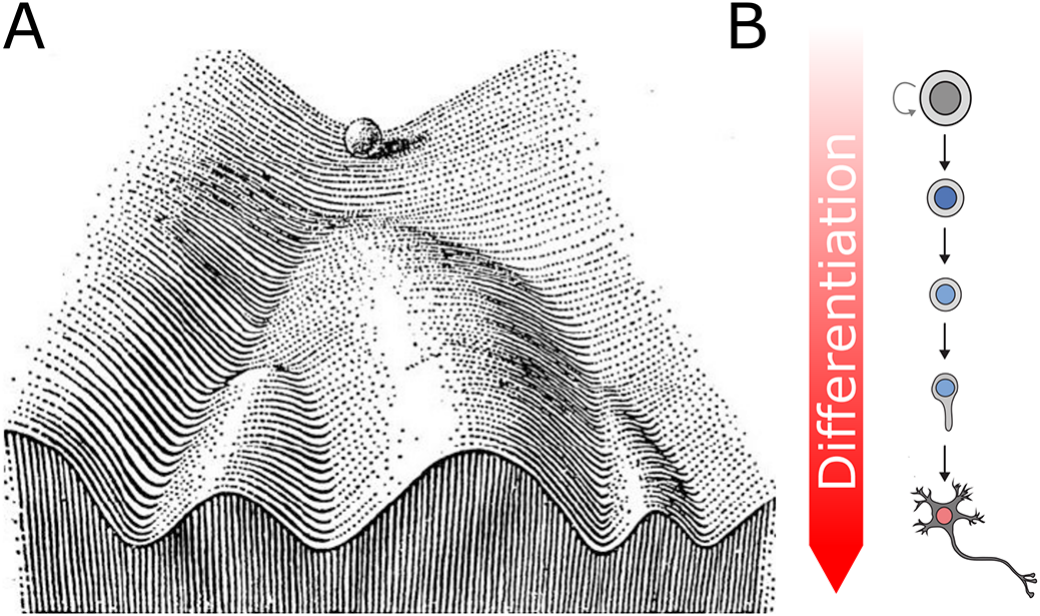
**Waddington's epigenetic landscape in neuronal differentiation.** (A) Waddington's original epigenetic landscape diagram representing developmental cell-fate trajectories that become more restricted as the cell differentiates [modified from Waddington (2014)]. (B) Schematic representing a neuronal lineage proceeding from neuronal stem cell to fully differentiated post-mitotic neuron.

In 2006 Takahashi and Yamanaka demonstrated that pluripotent cells could be derived from fibroblasts in culture by introducing a defined set of transcription factors (TFs) – thereby overriding the gene expression programmes responsible for maintaining the cells differentiated state (Takahashi and Yamanaka, 2006). This work provided the first evidence that cell fate could be reversible. However, in normal physiological conditions, once differentiated, cell fate is generally considered to be stable, with spontaneous reversion to previous states being extremely rare (although not unheard of, e.g. zebrafish heart regeneration; Jopling et al., 2010). This uni-directional process of differentiation is often communicated through the metaphor of Waddington's ‘epigenetic landscape’ (Waddington, 2014; Noble, 2015) (Fig. 1). In this model, cell fate is visualised as a series of valleys down which a ball (cell) rolls downwards from pluripotency at the top of the hill to fully differentiated states at the bottom. The presence of ridges within the landscape provides multiple routes for the ball to access (i.e. multiple developmental trajectories) leading to the eventual existence of multistable states that the ball may not move between. Importantly, in this analogy the ball representing a cell can only roll downhill (i.e. it cannot move against gravity). Whilst this creates a helpful picture to visualise the trajectory of cell fate through development, the molecular factors that shape the landscape are ill defined. Factors driving differentiation (pushing the ball downhill/towards particular trajectories) are increasingly well understood. However, the forces that prevent the metaphorical ball from rolling uphill (i.e. preventing dedifferentiation) are less well characterised.

**Fig. 2.**
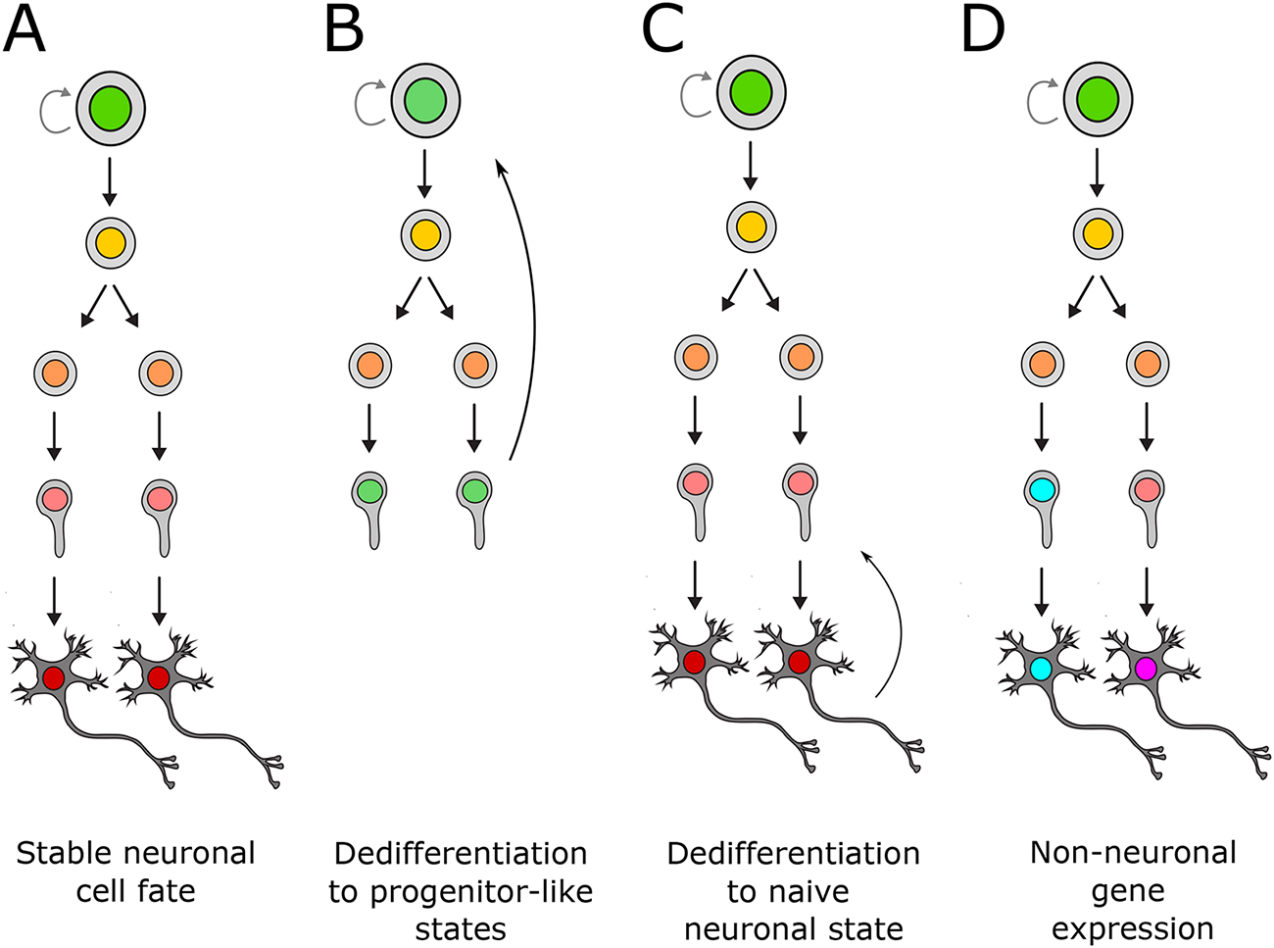
**Outcomes associated with disrupted maintenance of neuronal identity.** (A) Normal development and stable neuronal fate. (B) Complete dedifferentiation of immature neurons to neural stem-cell like fates. As seen with, e.g. Lola-N, Nerfin-1, or p53 knockdown in postmitotic cells. Can result in uncontrolled proliferation and tumour formation. (C) Dedifferentiation of mature neurons resulting in loss of neuronal subtype specification. Neurons revert to non-specified neuronal cell-fate, usually without proliferation, e.g. loss of terminal selectors. (D) Acquired expression of ectopic non-neuronal genes in post-mitotic neurons. Seen with loss of chromatin factors such as Mi-2/CHD4 or GLP/G9a.

Several studies in *Drosophila* have indicated that differentiated neurons may be reverted to stem-cell like states by removal of key transcription factors (Table 1). Loss of neuron-specific TFs *nerfin-1* and *Lola­* was sufficient to cause re-expression of neural stem-cell genes, as well as cell-cycle re-entry resulting in tumours formed of dedifferentiated cells (Froldi et al., 2015; Southall et al., 2014). Crucially, this process occurred following terminal cell division when neuronal markers were already visible (Fig. 2A,B). Therefore, whilst these transcription factors are not necessary to promote neuronal cell fate, they are required to maintain it. Similarly, knockdown of another transcription factor *midlife crisis* (*mdlc*), also causes ectopic expression of stem cell genes in *Drosophila* (Carney et al., 2013). In this case, expression of a conserved human orthologue (*RNF113A*) was sufficient to rescue the ectopic gene expression seen in *mdlc* mutants, indicating that neuronal cell-fate may be maintained by conserved mechanisms in mammalian neurons.

**Table 1.**
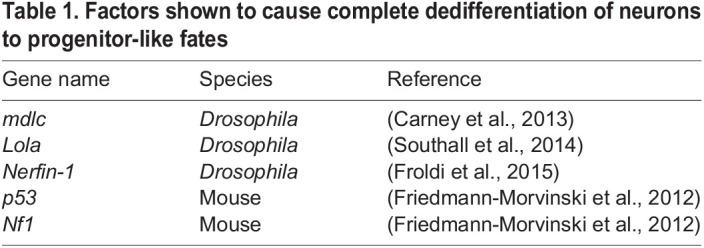
Factors shown to cause complete dedifferentiation of neurons to progenitor-like fates

Not all neurons in the brain have the same developmental origins, and consequently, have differences in the gene expression programmes that coordinate their functions. Interestingly, *Lola* mutations were only sufficient to cause dedifferentiation in neurons of the optic lobe (Southall et al., 2014), whereas loss of nerfin-1 resulted in loss of neuronal identity in all brain regions (Froldi et al., 2015). This discrepancy was explained by the fact that the key neuronal fate determinant, Prospero, continues to be expressed in the neurons of central brain and VNC lineages, but not in the optic lobe. This observation highlights that there may not be one defined pathway or defined set of molecular factors that maintains overall neuronal identity in all neurons, and there are likely to be further lineage specific differences. It has also been observed that although differentiated neurons exhibiting standard neuronal markers may be induced to dedifferentiate through loss of Lola or Nerfin-1, neurons may become more refractory to dedifferentiation following maturation, since in multiple cases phenotypes associated with loss of neuronal identity became more resistant as neurons became more mature (Friedmann-Morvinski et al., 2012; Southall et al., 2014). Therefore, there may be as yet unidentified differentiation checkpoints following which the mechanisms that maintain neuronal cell-fate become more robust.

Intuitively, TFs that cause dedifferentiation when depleted may be thought to act specifically as repressors of cell cycle and stem-cell associated genes. However, profiling the genome-wide binding sites of Lola and Nerfin-1 indicates that these factors bind to both cell-cycle/stem cell genes, as well as neuronal genes (Southall et al., 2014; Vissers et al., 2018). This is consistent with the observation that as well as re-activating stem cell gene expression, these mutants also lose expression of neuronal markers. Therefore, rather than acting solely as repressors, the function of these TFs is likely to be modulated in a context specific manner. It is interesting to speculate that this locus-specific repression/expression activity may be a necessary feature of TFs that maintain neuronal fate to confer robust bistability as described by Waddington's epigenetic landscape (i.e. they direct the ball towards a single valley while simultaneously imposing a ‘gravitational’ force that the ball cannot move against). Identifying co-factors that confer locus-specific repression or upregulation will be key to fully understanding how these proteins maintain differentiated neuronal states.

In contrast to either *nerfin-1* or *lola* mutants, loss of *mdlc* was not sufficient to cause cell-cycle re-entry in neurons. Therefore, while loss of neuronal cell fate may be accompanied by cell-cycle re-entry, these two processes are not necessarily intrinsically linked. Conversely, in mouse brains or cultured cells that are mutant for *E2F1* (a well characterised regulator of cell cycle gene expression), neurons are able to proliferate, but do not lose neuronal markers, thereby lending support for the idea that neuronal cell fate and cell-cycle exit are regulated via distinct mechanisms (Wang et al., 2007). However, the fact that Lola binds to cell-cycle gene loci, and presumably contributes to their repression suggests that some factors that act to maintain neuronal identity may also reinforce cell-cycle exit in post-mitotic cells. Factors such as Lola or Nerfin may act to integrate cell cycle exit and initiation of neuronal-fate maintenance mechanisms, which are then more robustly conferred by subsequent regulatory events. Further evidence that cell-cycle regulation and cell-fate maintenance may be interrelated comes from the observation that Retinoblastoma (Rb) family proteins (known for suppressing cell cycle gene expression) are required for maintaining the differentiated state of retinal cells in *Drosophila* (Nicolay et al., 2010). Double knockdown of *Drosophila Rb* orthologue *Rbf* in retinal cells along with hippo pathway components caused dedifferentiation and uncontrolled proliferation. While these cells lost their specific photoreceptor identity, they did not lose all neuronal markers indicating that they had reverted to uncommitted retinal cell fate without losing overall neuronal identity. Interestingly, if proliferation was blocked, the cells were still seen to dedifferentiate, implying that the role of Rbf in maintaining mature photoreceptor fate is independent of its cell-cycle function.

## Maintenance of neuronal subtype specification by terminal selector genes

The maintenance of neuronal gene expression programmes requires not only the long-term repression of non-neuronal genes, but also the sustained transcription of genes that are required for neuronal function. Following specification of neuronal subtypes, transcription of key genes that coordinate that cell's specific activities (such as neurotransmitter receptors and enzymes required for their synthesis), are stably expressed for the lifetime of the neuron. The specification of neuronal subtype identity is determined by lineage specific transcription factors during differentiation. However, these genes, known as terminal selectors, continue to be expressed in mature neurons suggesting that they are also required for maintenance of neuronal identity.

In support of the idea that terminal selectors are also required for maintenance of neuronal subtype specification, several studies have reported that loss of these genes results in loss of markers associated with the identity in question (Fig. 2C). (For comprehensive overview of terminal selectors involved in neuronal subtype maintenance, see Deneris and Hobert (2014). Much of this work has been conducted using *C. elegans*, in which the well-defined nervous system and genetic tractability have facilitated the study of the properties of anatomically and functionally characterised groups of neurons. For example, the terminal selector *ast-1* specifies the dopaminergic subtype, and moreover; loss of *ast-1* in mature dopaminergic neurons was sufficient to cause these cells to lose their dopaminergic identity (Flames and Hobert, 2009). Similar observations have been made for glutamatergic neurons (Serrano-Saiz et al., 2013), and gustatory neurons (O'Meara et al., 2010). Likewise, further invertebrate models have shown similar actions of terminal selectors in conferring long-term maintenance of neuronal specification (Hsiao et al., 2013; März et al., 2013). Transcription factors with similar functions have also been identified in mouse, indicating a broadly conserved mechanism for maintaining neuronal specification via sustained expression of sequence-specific terminal selectors (Magno et al., 2011; Schmidt et al., 2009; Stott et al., 2013).

Since there may be many hundreds of unique neuronal subtypes in a complex nervous system, identifying the transcription factors that are associated with their maintenance is a significant challenge. Advances in technologies allowing for molecular profiling with increased temporal and spatial resolution are being used to great effect in the effort to identify these factors. For example, the application of Targeted DamID to discrete neurotransmitter expressing cell types was used to catalogue transcription factor expression at different developmental stages in *Drosophila*, identifying 86 unique transcription factors that were expressed in individual neurotransmitter types, which could be further subclassified into factors that had sustained expression in all life stages (Estacio-Gómez et al., 2020). Even greater resolution is achieved by the application of single-cell sequencing technologies. For example, one recent study focusing on the fly visual system identified ten transcription factors, which when expressed in various combinations specified the (approximately) 200 cell types in the visual system (Özel et al., 2022). Importantly, expression of these genes was sustained post-mitotically, and targeted depletion of individual factors was sufficient for conversion of developing neurons to other subtypes in a predictable manner.

Expression of terminal selector genes that maintain subtype identity is often initiated by transiently expressed transcription factors at early stages of neurogenesis, (for example in the case of *C. elegansttx-3* and *ceh-10* activation by transiently expressed factors in precursor cells) (Bertrand and Hobert, 2009). Therefore, there must be mechanisms in place to maintain the stable expression of these critical factors throughout neuronal lifespans. This phenomenon may be partly explained by positive feedback loops in which expression of a transcription factor gene is reinforced by direct association with its own locus, a process known as autoregulation. Autoregulation is a common regulatory mechanism, first identified in bacteriophage (Ptashne et al., 1976), and has been documented for neuronal terminal selector genes in *C. elegans* (Etchberger et al., 2007; Sarafi-Reinach et al., 2001; Way and Chalfie, 1989) and mouse (Liu et al., 2010). While many of these studies showed association in *cis* of transcription factors with their own loci, more recent studies have further dissected the functional consequences of these autoregulatory loops by taking advantage of genome editing. One such study demonstrated that targeted mutations of a putative autoregulatory enhancer in the *che-1* locus of *C. elegans*, caused salt-sensing ASE-neurons to fail to properly specify by not reaching threshold levels of expression, and also fail to maintain ASE-fate (Leyva-Díaz and Hobert, 2019). Similar results were also observed in mouse for the *Ptf1a* gene, required for proper specification of ‘itch-circuit’ neurons (Mona et al., 2020). Therefore, autoregulation is an important conserved mechanism for ensuring that neuronal subtype specification is effectively ‘locked-in’ over long timescales.

Despite the clear importance of autoregulation in maintaining neuronal subtype identity, questions remain regarding the extent to which autoregulation is sufficient for long-lasting maintenance of identity. A recent study further investigating the role of CHE-1 autoregulation of ASE identity concluded that a key feature of the feedback loop was increased preference for CHE-1 to bind its own locus (over the 500-1000 other che-1 target genes) (Traets et al., 2021). By this mechanism ASE-neurons were able to tolerate molecular fluctuations in CHE-1 levels, showing resilience to transient CHE-1 knockdowns, (whereas extended loss of CHE-1 resulted in irreversible loss of neuronal identity). Interestingly, the increased preference for CHE-1 to bind its own locus was mediated partly by a cis-regulatory element distal from the *che-1* promoter, suggesting that further co-factors may be required to reinforce terminal selector expression. Besides autoregulation, there may be other mechanisms that operate redundantly or uniquely for different factors to ensure sustained neuronal gene expression. For instance, recent research has shown that conserved homeobox (HOX) family transcription factors, which are best known for their role in developmental patterning, are required to maintain cholinergic identity in *C. elegans* motor neurons (Feng et al., 2022). HOX genes were shown to utilise a feed-forward mechanism (i.e. promoting the activation of a further gene that in turn activates cholinergic target genes), which reinforces robust expression of motor neuron specific transcripts, as well as employing autoregulation to sustain their own expression.

### Suppression of lineage-inappropriate gene expression in neurons by chromatin states

Fully differentiated neurons house the entire genome of an organism within their nuclei. Of this full complement of DNA, only some genes and regulatory elements are needed for neuronal function. This includes broadly expressed housekeeping genes that are necessary for cell survival, as well as a more restricted set of neuronal genes required for the specific biology of this highly specialised cell-type. Ectopic expression of non-neuronal genes is likely to cause deleterious effects; therefore, the expression of these genes must be suppressed throughout the lifetime of the neuron.

We are now beginning to understand the nature of repressive chromatin states in neurons and how they are established and maintained. Assaying chromatin accessibility provides a simplistic, but informative overview of loci that are involved in transcription (i.e. open/accessible) or repressed (closed/inaccessible) (Minnoye et al., 2021). Recent technological advances that allow for spatial resolution of chromatin accessibility within complex tissues (i.e. the brain), have been informative in understanding the nature of the chromatin transitions that accompany differentiation. One such study exploited the selective labelling *in vivo* of accessible loci by a transgene expressed DNA adenine methylase (Dam) to show that overall chromatin accessibility becomes increasingly restricted as neural lineages differentiate more fully (Aughey et al., 2018). Therefore, the regulatory elements that have the potential to coordinate gene-regulatory networks become more restricted and the cell-fate choices and transcriptional outcomes of the neuron become fewer. This narrowing of transcriptional diversity, whilst an intuitive result, had previously only been speculated at in *in vivo* tissues. However, examination of single-cell gene counts from multiple organisms indicate that this feature is likely a hallmark of differentiated cells as their developmental potential is constrained (Gulati et al., 2020). There is some evidence that this restriction of plasticity is established partly by terminal selector genes in *C. elegans* (Patel and Hobert, 2017). Ectopic expression of CHE-1 in differentiated non-ASE neurons was insufficient to cause expression of its ASE-neuron target genes, however, when terminal selectors of those neurons were knocked down, ectopic CHE-1 was able to promote ASE-neuron identity. This restriction of neuronal plasticity by terminal selectors was suggested to be conferred by altering the chromatin state of target promoters via the action of H3K9 methyltransferases.

Chromatin accessibility assays may provide valuable insights into neuronal epigenetic landscapes and can be used to infer specific transcription factors involved in differentiation. However, these assays do not specifically reflect the nature of the changes to the underlying chromatin that coordinate different transcriptional responses. More targeted descriptive studies have shed light on these processes. For example, more complete maps of major histone modifications have been produced for neuronal lineages, either by assessing histone modifications with ChIP-seq from sorted populations of cells (Abdusselamoglu et al., 2019; Södersten et al., 2018), or more recently by single-cell approaches such as single-cell CUT&TAG (Bartosovic et al., 2021).

These descriptive studies will continue to be useful in understanding the nature and extent of the chromatin changes occurring in fully differentiated cells. In parallel, more nuanced chromatin state modelling is further advancing our understanding of the mechanisms by which repressive and active states are established and maintained. Integration of multiple chromatin features allows for the identification of discrete chromatin state models in which the entire genome can be partitioned into distinct sub-types that reflect unique gene-regulatory environments (Ernst and Kellis, 2010; Filion et al., 2010). Applying these methods to neuronal lineages in *Drosophila* showed that five major chromatin states underlie gene regulation which undergo dynamic transitions as neuronal lineages differentiate (Marshall and Brand, 2017). Using this approach, a number of surprising features are identified. Firstly, few stem-cell genes appear to be repressed by polycomb-associated states, despite the prevailing view of polycomb proteins as major repressive factors. Rather, polycomb appeared to mostly be important in repression of genes in specific neuronal lineages rather than being important for maintaining the distinction between progenitor and differentiated cell types. Instead, most repression appeared to be governed by HP1-associated chromatin states, while some genes are also repressed by a trithorax-associated repressive state. A more recent analysis, which integrated further datasets, concluded that there are as many as eight principal states (Delandre et al., 2022 preprint). This analysis highlighted the potential for a ‘yellow’ (Swi/Snf – repressive) chromatin state involvement in the silencing of cell-cycle genes in mature neurons. Another interesting feature of chromatin state models is the presence of a ‘black’ chromatin state that does not correlate with any major complexes or histone marks other than an enrichment for linker histone H1. This presumptive silent chromatin state is not associated with known repressive complexes, so it is unclear whether it is actively repressed or merely silent by virtue of the absence of activating transcription factors. However, following differentiation, much of the ‘black’ state transitions to HP1-enriched states suggesting that once fully mature, more robust mechanisms are put into place for the long-term repression of non-neuronal genes. Similar analysis of chromatin states in other tissues would shed light on whether these transitions are a neuron-specific feature, or a hallmark of all differentiated tissues.

## Chromatin modifying complexes preventing dedifferentiation

As previously discussed, several sequence-specific transcription factors are required to maintain neuronal cell-fate in neurons. However, it remains unclear what mechanisms act downstream of these factors to ensure robust lineage identity. It is likely that transcription factors associate with chromatin modifying complexes that affect stable repression/activation at target loci via histone modifications and/or nucleosome remodelling. Some of the changes enacted by these enzymes are evident from the molecular profiling experiments described previously. However, identifying the specific complexes responsible remains a major challenge for understanding the exact processes by which neuronal states are maintained.

Chromatin state models suggest that polycomb proteins are responsible for neuronal subtype specification rather than broad repression of non-neuronal transcriptional programmes. In support of this idea, functional studies *in vivo* have demonstrated a role for polycomb in maintaining neuronal specification. Firstly, in *C. elegans*, knockdown of Polycomb repressive complex 1 (PRC1) components was sufficient to cause stochastic loss of neuronal identity in several neuronal subtypes (Bordet et al., 2022). It was shown that PRC1 is required for consistent expression of terminal selector genes, the loss of which predictably results in loss of subtype identity. Similarly, loss of Polycomb repressive complex 2 (PRC2) in mouse neurons resulted in global loss of H3K27me3, and subsequent loss of dopaminergic and serotonergic identity (Toskas et al., 2022). In this case it is unclear whether PRC2 is involved in terminal selector gene regulation as with PRC1 in *C elegans*, however, this work demonstrates that polycomb complexes are likely to have broadly conserved roles in maintaining neuronal subtype identity.

Chromatin modifying complexes have also been implicated in the maintenance of overall neuronal lineage fate. Loss of SWI/SNF in neural stem cells in *Drosophila* causes transit-amplifying progeny to revert to stem-cell like fates, resulting in neoplastic growth and tumour formation (Eroglu et al., 2014). While this phenotype is similar to that seen with *nerfin-1* or *Lola* mutants, it is unclear whether SWI/SNF continues to ensure neuronal fate following cell-cycle exit and terminal differentiation. To date, no single complex has been demonstrated to result in dedifferentiation of mature neurons. This may be because the chromatin states they confer are stable for very long timescales or may be indicative of more complex multi-factor mechanisms. However, it is also difficult to assess the function of these complexes through loss of function experiments since many have roles in core transcriptional machinery or other gene regulatory processes that are essential for cell viability. Further study will be required to determine which complexes act to prevent dedifferentiation and maintain neuronal fate.

## Chromatin modifying complexes suppressing lineage-inappropriate gene expression

Major lineage commitments occur during the early stages of embryogenesis, most notably at gastrulation at which point three distinct germ layers are specified. Genes that are specific to a particular lineages' specialised cell-types are fully repressed permanently in other lineages. Therefore, neuronal identity must be maintained not only by repression of progenitor genes, but also by repressing the expression of lineage inappropriate transcription (Fig. 2D) (Table 2). Ectopic gene expression is likely to be deleterious to neuronal function, and such aberrant gene expression has been implicated in a variety of neurodevelopmental disorders (Bonefas and Iwase, 2022). Mutations of various chromatin factors is sufficient to result in non-neuronal gene expression in brain tissue, however, since progenitor cells are affected as well as neurons, it is currently difficult to say what contribution these genes have to maintaining gene repression in differentiated post-mitotic cells (Ben-Shachar et al., 2009; Velasco et al., 2010).

**Table 2.**
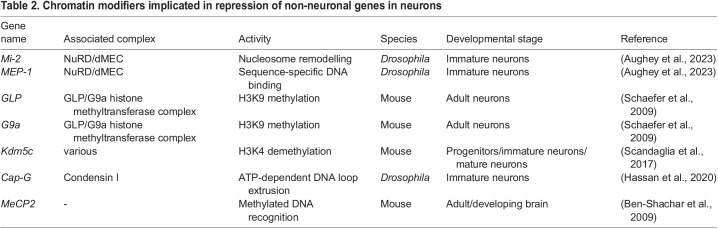
Chromatin modifiers implicated in repression of non-neuronal genes in neurons

Recently, the conserved chromatin remodeller Mi-2/CHD4 was shown to be required for the repression of such ectopic gene expression in differentiating neurons in *Drosophila* (Aughey et al., 2023). Loss of Mi-2 resulted in broad expression of genes that were not normally associated with neuronal transcriptomes. In contrast to the factors discussed earlier, the mis-expressed genes were not cell-cycle and neuronal stem-cell genes, but genes that are usually expressed in completely separate organ systems, particularly the germline. Mi-2 is a core subunit of the highly conserved NuRD (nucleosome remodelling and deacetylase) complex, which possesses both ATP-dependent nucleosome remodelling, as well as histone deacetylation activities via separate enzymatic subunits. The histone deacetylation components of the complex were not required for the suppression of lineage-inappropriate gene expression, indicating that nucleosome positioning is a key mechanism in maintaining neuronal transcriptome identity by repressing non-neuronal gene expression.

Interestingly, misexpression of germline genes in Mi-2 knockdown neurons was only observed in immature neurons in the larval brain and not fully mature adult neurons, indicating that nucleosome repositioning at these loci is less important after a stable epigenetic state has been established. Similar results have been observed for mutants of the histone demethylase, Kdm5c, in mouse brains. Kdm5c mutants display ectopic germline gene expression in mutants, associated with elevated H3K4 trimethylation (Scandaglia et al., 2017). However, this effect became less pronounced as neurons matured, and reintroduction of kdm5c was insufficient to rescue neuronal germline gene expression, although kdmc5 expression was still required for repression of spurious expression at some loci. These data indicate that appropriate histone methylation during development ensures stable repression on non-neuronal genes, but that this activity continues to play a role in adult neurons albeit to a lesser extent.

Expression of non-neuronal genes was also observed when the H3K9 methyltransferase GLP/G9a complex was disrupted specifically in mouse neurons (Schaefer et al., 2009). Knockout of either *GLP* or *G9a* were associated with a reduction of the H3K9me2 repressive mark in euchromatic regions. In contrast to *Kdm5c* or *Mi-2* knockdowns, robust expression of non-neuronal genes was observed when GLP or G9a were depleted specifically in mature adult neurons. Therefore, H3K9 methylation appears to require more active ongoing maintenance in neurons than some other epigenetic features to stably repress transcription.

Similar results were also observed when the condensin complex component, Cap-G was depleted in *Drosophila* neurons (Hassan et al., 2020). Genes annotated with non-neuronal gene ontologies such as ‘midgut development’ were upregulated, along with downregulation of genes involved in neuronal function. Interestingly, this protein is best characterised in the condensation of mitotic chromatin during cell division by ATP-dependent loop extrusion. It is unclear whether the full condensin complex is involved in suppressing non-neuronal genes in post-mitotic cells, or whether Cap-G acts in an independent role, however, it is intriguing to speculate that post-mitotic control of DNA conformation via condensin or related proteins may also be involved in maintaining neuron-appropriate transcriptomes.

Identifying unique or enriched chromatin features in neuronal nuclei may provide clues to the mechanisms involved in the unique long-term maintenance of transcriptional states in neurons. For example, neuronal DNA in mammals contains a unique class of cytosine methylation distinct from widespread CG methylation. This non-CG methylation is enriched in CA dinucleotides and can reach levels comparable to CG methylation (Guo et al., 2014). Cytosine methylation is recognised by Methyl-CpG-binding protein 2 (MeCP2) (Lagger et al., 2017; Lewis et al., 1992), which may have repressive or activating functions (Chahrour et al., 2008). Similarly to the previously described factors, *MeCP2* mutants are associated with expression of non-neuronal genes in the brain, indicating that cytosine methylation may be important for maintaining gene repression in neurons (Ben-Shachar et al., 2009). However, it is unclear whether ectopic expression is seen in mature neurons or only as a consequence of MeCP2 loss in progenitors, although neurological phenotypes can be rescued by re-introduction of MeCP2 only in adult brains (Guy et al., 2007).

## Contribution of disrupted neuronal identity maintenance to pathological states

Loss of neuronal identity can have severe phenotypic consequences in model organisms. Therefore, it is unsurprising that disruption of the epigenetic status of neurons with associated signs of dedifferentiation or lineage inappropriate gene expression has been implicated in a range of pathologies that affect the human nervous system. Mutations in critical chromatin modifying enzymes that confer repression of non-neuronal gene expression can result in severe neurodevelopmental disorders in human patients. For example, mutations in *G9a/GLP* methyltransferases, or *KDM5C* demethylases result in complex developmental phenotypes with mental retardation (Jensen et al., 2005; Kleefstra et al., 2006).

Neuronal dedifferentiation has been implicated in the aetiology of certain human cancers. In a mouse model, neuron specific knockdown of two genes (*p53* and *Nf1*) known to be involved in human glioblastomas was sufficient to produce proliferating gliomas (Friedmann-Morvinski et al., 2012). The cells in these tumours exhibited a loss of neuronal markers and an increase in progenitor markers; features that became more evident as the tumours progressed. Interestingly, when increasingly mature neurons were targeted in the mouse model, they appeared to be more resistant to dedifferentiation and tumour formation. This observation lends further support to the idea that the mechanisms that maintain neuronal identity become more robust as neurons progress through maturation.

While these results are strongly suggestive of the possibility of dedifferentiated neurons being the point of origin for some brain tumours, the extent to which this actually occurs in humans is still not fully understood (Fan et al., 2019). The ability of neuronally derived tumours to access the growth-regulatory gene expression programmes of its progenitors offers a significant advantage to proliferation, whilst continued expression of neuronal genes is likely to be growth-inhibiting. On the other hand, some brain tumours exhibit neuron-like activities such as synapse formation and calcium oscillations which promote malignancy, suggesting that full dedifferentiation may not always be beneficial to tumour growth (Hausmann et al., 2023; Venkataramani et al., 2019; Venkatesh et al., 2019). Therefore, understanding how the mechanisms that maintain neuronal identity may be overcome in healthy neurons may help to better understand tumour aetiology leading to malignant growth.

A growing body of evidence points towards the loss of mechanisms conferring robust neuronal identity being a major contributor to neurodegenerative disorders. For example, there is extensive evidence that in Alzheimer's disease neurons may begin to display dedifferentiation-like characteristics such as expression of cell-cycle genes (Arendt, 2012), while *in vitro* differentiated neurons from Alzheimer's patients appear less capable of maintaining differentiated cell fates (Mertens et al., 2021). Rather than resulting in aberrant proliferation, the re-expression of cell cycle genes in neurodegenerative disorders is associated with increased likelihood of undergoing apoptosis, which may be a contributing factor to the neurodegenerative symptoms displayed by Alzheimer's patients (Busser et al., 1998; Frade and Ovejero-Benito, 2015).

The consequences of the disruption of neuronal maintenance may also be apparent in ageing neurons generally, without being associated with a specific pathological state. Studies in model organisms have suggested that a major contributor to ageing is the loss of cell's epigenetic status (Haithcock et al., 2005; Larson et al., 2012; Yang et al., 2023), and altered epigenomes have been reported in ageing neuronal tissue (Rodrigues et al., 2014). It remains unclear whether loss of epigenetic states is a major cause of age-related symptoms in humans, or rather a consequence of other age-related deterioration. However, it is likely that gradual erosion of chromatin states that ensure robust gene expression/repression results in sub-optimal ability of neurons to perform their given role within the nervous system, even if symptoms fall short of diagnosable disease.

### Outstanding questions and conclusions

It is increasingly clear that the maintenance of neuronal states is an active process that continues throughout the lifetime of the cell to prevent aberrant gene expression leading to pathological states. However, several outstanding questions must be addressed to fully understand the maintenance of neuronal gene expression and associated cell-fate.

#### When are the critical periods during neuronal maturation during which stable chromatin states are established?

The loss of transcription factors required to prevent dedifferentiation or ectopic expression of lineage-inappropriate genes may produce phenotypes only in newly born neurons (Aughey et al., 2023; Friedmann-Morvinski et al., 2012; Southall et al., 2014). Similarly, ectopic expression of terminal selectors is only sufficient to redirect cell fate before terminal differentiation occurs (Patel and Hobert, 2017). This suggests that checkpoints exist after which transcriptome stability is more robustly conferred by a lasting epigenetic environment. Greater temporal dissection of these critical periods will help to understand which molecular factors are required and how they relate to the wider process of neuronal maturation. Increased uptake of single-cell approaches such as single-cell CUT&Tag will help to better characterise the trajectory of histone modifications in differentiating neuronal lineages (Bartosovic et al., 2021). However, improved technologies may need to be developed to better target experimental interventions to more restricted spatial/temporal windows in animal brains *in vivo* to obtain complete mechanistic insight into these processes.

#### How are repressive chromatin states maintained throughout the life of neurons, and is this an active process?

**Fig. 3.**
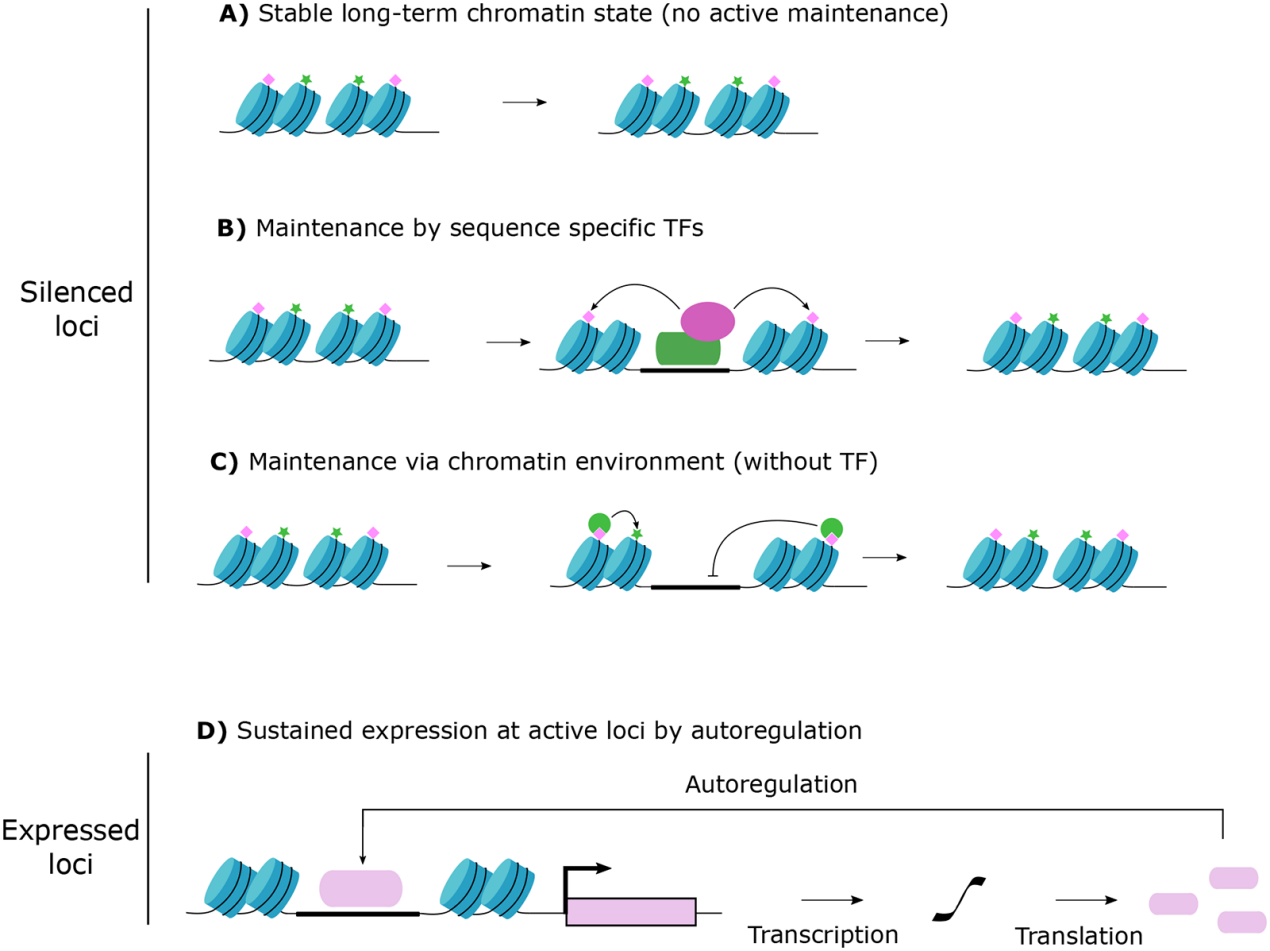
**Potential strategies for maintaining neuron-appropriate transcriptomes in mature neurons.** (A) Epigenetic modifications (green stars/pink diamonds) established during maturation may be stable for neuronal lifespan without the requirement for active maintenance of repressive environment. (B) Continued occupancy or surveillance by sequence-specific transcription factors (green) may be required to recruit chromatin modifiers (purple) for ongoing repression at non-neuronal loci. (C) Epigenetic states may be self-reinforcing at suppressed loci without the requirement the sequence-specific transcription factors that were required for their establishment in immature neurons. (D) Sustained active gene expression of genes required for neuronal state maintenance by autoregulation (e.g. terminal selectors).

Once repressive epigenetic environments have been initialised during neuronal maturation, how are these states maintained over long timescales? It is possible that the gradual loss of epigenetic markers seen during ageing is simply due to entropy of chromatin that has no active mechanisms to restore histone modifications at loci where these marks have been lost over time (Fig. 3). Alternatively, the loss of fidelity of the mechanisms that maintain repressive states could be explained by other environmental factors such as accumulating DNA damage. For at least some loci, loss of enzymes that deposit repressive histone marks is sufficient to cause de-repression of non-neuronal genes, but it remains unclear whether this is consistent for all repressed loci, and which modifications must be continually maintained.

As noted earlier, the fact that many factors responsible for suppressing the expression of non-neuronal genes also promote neuronal gene expression suggests that there may be a constant pool of sequence-specific transcription factors that may be able to bind to loci that become accessible over time due to loss of repressive marks, thereby promoting the re-establishment of the appropriate histone marks or nucleosome positioning. However, it remains unclear whether any necessary co-factors that modify the activity of these factors in a context-specific manner continue to be expressed.

It will be necessary to answer these questions to gain a complete understanding of how neuronal transcriptomes and associated cell-fate is maintained in mature tissues. With improved knowledge of these mechanisms, targeted development of therapeutics to treat disorders involved in misregulation of neuronal transcription may be possible. Whilst this Review has focused on neurons, the principles by which differentiated transcriptomes are maintained are likely to be shared across many tissues outside of the nervous system.
